# Supplementary material for: KCNQ2 Selectivity Filter Mutations Cause Kv7.2 M-Current Dysfunction and Configuration Changes Manifesting as Epileptic Encephalopathies and Autistic Spectrum Disorders
Source: Cells. 2022 Mar 5;11(5):894. doi: 10.3390/cells11050894 (PMC8909571; doi:10.3390/cells11050894)

Supplemental Figure S1. The total filters for the KCNQ2 protein expression on cell membranes are demonstrated and in the Figure 2D.

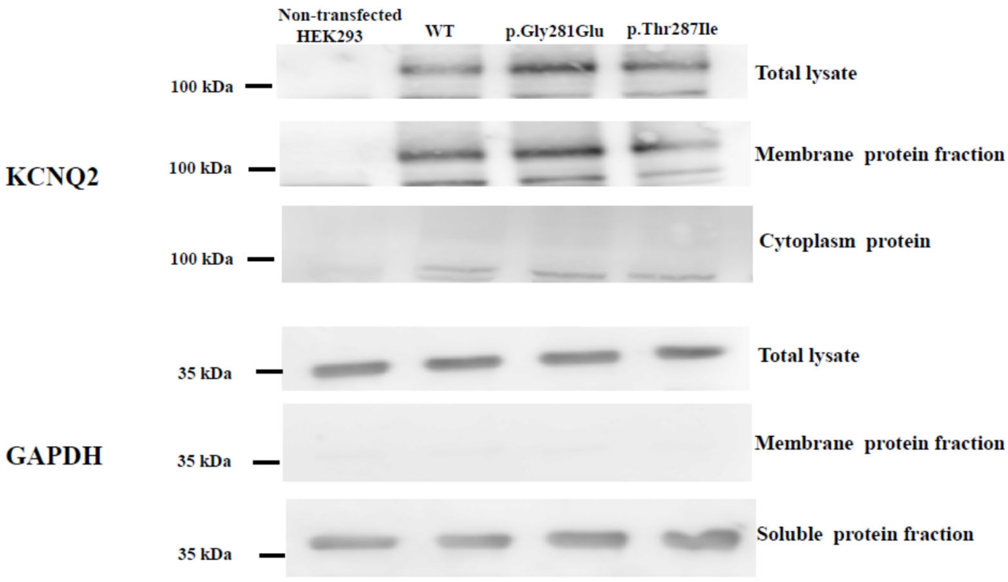

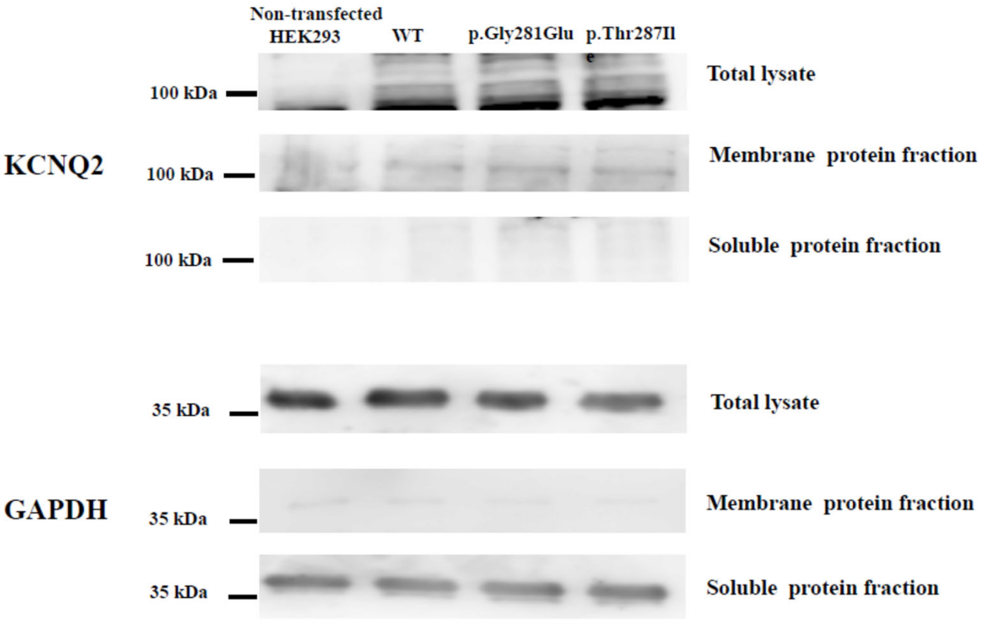

Supplement: Supplementary file 1 [file cells-11-00894-s001.zip › cells-1445147-supplementary/Supplementary Figure S1.pdf]
