# Supplementary material for: KCNQ2 Selectivity Filter Mutations Cause Kv7.2 M-Current Dysfunction and Configuration Changes Manifesting as Epileptic Encephalopathies and Autistic Spectrum Disorders
Source: Cells. 2022 Mar 5;11(5):894. doi: 10.3390/cells11050894 (PMC8909571; doi:10.3390/cells11050894)
Supplement: Supplementary file 1 [file cells-11-00894-s001.zip › cells-1445147-supplementary/Supplementary Table S1.pdf]

Supplementary Table S1. Currents and $V_{1/2}$ in homomeric and heteromeric transfected HEK293 cell in the variants of p.Gly281Glu, p.Thr287Ile, and p.Gly281Glu.

	N	Currents at -10 mV (pA) (mean±SD)	Currents at 0 mV (pA) (mean±SD)	Currents at +10 mV (pA) (mean±SD)	Currents at +20 mV (pA) (mean±SD)	Currents at +30 mV (pA) (mean±SD)	Currents at +40 mV (pA) (mean±SD)
KCNQ2 WT (2 ug) [#]	22	596.5 ± 121.5	769.3 ± 119.3	954.1 ± 121.5	1145.8 ± 117.9	1333.9 ± 120.8	1534.5 ± 141.9
p.Gly281Glu (2 ug)	8	**480.9 ± 101.5**<br>**t(30) = 3.51, *p* = 0.001**	**600.0 ± 96.6**<br>**t(30) = 4.70, *p* < .001**	**721.8 ± 89.3**<br>**t(30) = 6.07, *p* < .001**	**852.8 ± 91.6**<br>**t(30) = 7.25, *p* < .001**	**979.6 ± 97.1**<br>**t(30) = 8.78, *p* < .001**	**1109.4 ± 112.6**<br>**t(30) = 8.76, *p* < .001**
p.Thr287Ile (2 ug)	10	**505.3 ± 56.25**<br>**t(30) = 2.25, p = .032**	**649.9±85.7**<br>**t(30) = 2.84, p = .008**	**789.5±92.9**<br>**t(30) = 3.80, p = .001**	**937.7 ± 99.1**<br>**t(30) = 4.85, p < .001**	**1087.0 ± 107.3**<br>**t(30) = 5.54, p < .001**	**1240.1 ± 122.2**<br>**t(30) = 5.66, p < .001**
p.Pro285Thr (2 ug)	10	**465.2 ± 64.0**<br>**t(30) =3.20, *p* = .003**	**599.7±76.4**<br>**(t(30) =4.11, *p* < .001**	**747.5±93.7**<br>**(t(30) =4.76, *p* < .001**	**890.9 ± 110.5**<br>**(t(30) =5.78, *p* < .001**	**1035 ± 110.6**<br>**t(30) =6.65, *p* < .001**	**1173.3 ± 126.5**<br>**t(30) =6.89, *p* < .001**
KCNQ2 WT + p.Gly281Glu (1ug:1 ug)	8	534.0 ± 75.3<br>t(30) =1.43, *p* = .164	**679.1±61.0**<br>**t(30) = 2.14, *p* = .041**	**843.4±63.6**<br>**t(30) = 2.57, *p* = .015**	**987.1 ± 51.0**<br>**t(30) = 3.86, *p* < .001**	**1137.6 ± 64.3**<br>**t(30) = 4.59, *p* < .001**	**1323.1 ± 81.0**<br>**t(30) = 4.18, *p* < .001**
KCNQ2 WT + p.Thr287Ile (1ug:1 ug)	10	548.3 ± 62.37<br>t(30) =1.18, *p* = .248	696.0±76.3<br>t(30) =1.78, *p* = .086	**837.5±88.0**<br>**t(30) =2.72, *p* = .011**	**1007.6 ± 95.3**<br>**t(30) =3.25, *p* = .003**	**1164.3 ± 112.8**<br>**t(30) =3.75, *p* < .001**	**1323.5 ± 99.9**<br>**t(30) =4.23, *p* < .001**
KCNQ2 WT + p.Pro285Thr (1ug:1 ug)	10	540.9 ± 76.04<br>t(18) = 1.33, *p* = 0.194	687.2±90.3<br>t(18) = 1.93, *p* = 0.063	858.8±102.6<br>**t(18) = 2.15, *p* = 0.040**	1030.2 ±122.6<br>**t(18) = 2.54, *p* = 0.016**	1206.8 ± 152.5<br>**t(18) = 2.54, *p* = 0.016**	**1385.4 ± 163.6**<br>**t(30) = 2.63, p = 0 .013**
KCNQ2 WT + *KCNQ3* WT (1ug:1 ug) [$]	10	934.6 ± 151.96	1218.5±184.3	1530.9±236.7	1843.1 ± 290.9	2168.3 ± 330.6	2474.3 ± 395.3
KCNQ2 WT + p.Gly281Glu + *KCNQ3* WT (0.5 ug: 0.5 ug: 1 ug)	8	**673.4 ± 87.4**<br>**t(18) =4.31, *p* = 0.001**	**914.1±120.9**<br>**t(18) =4.02, *p* = 0.001**	**1179.1±129.6**<br>**t(18) =3.76, *p* = 0.002**	**1481.4±116.6**<br>**t(18) =3.30, *p* = 0.005**	**1824.5 ± 117.5**<br>**t(18) =2.79, *p* = 0.013**	**2120.0 ± 65.9**<br>**t(18) =2.49, *p* = 0.024**
KCNQ2 WT + p.Thr287Ile + *KCNQ3* WT	10	818.1 ± 185.8	**1012.8±211.6**	**1244.7±265.0**	**1435.4±218.4**	**1667.1 ± 225.3**	**1865.0 ± 155.9**

(0.5 ug: 0.5 ug: 1 ug)		t(18) = 1.50, *p* = 0.150	**t(18) = 2.38, p = 0.029**	**t(18) = 2.63, p = 0.017**	**t(18) = 3.43, p = 0.003**	**t(18) = 3.86, p = 0.001**	**t(18) = 4.24, P < .001**
KCNQ2 WT + p.Pro285Thr + *KCNQ3* WT	10	820.9 ±133.1	1065.8±144.9	**1321.1±165.1**	**1570.0±190.6**	**1803.6 ± 223.6**	**2038.8 ± 251.4**
(0.5 ug: 0.5 ug: 1 ug)		t(18) =1.78, *p* = 0.092	t(18) =2.06, *p* = 0.054	**t(18) =2.30, *p* = 0.034**	**t(18) =2.48, *p* = 0.023**	**t(18) =2.89, *p* = 0.010**	**t(18) =2.94, *p* = 0.009**

Bold fonts indicate $p < 0.05$.

WT, wild type; SD, standard deviation. The data were then fit to a Boltzmann distribution of the following form: $G/Gmax = 1/(1 + exp[(V-V½)/dx])$.

[#] The currents in the homomeric transfected variants and heteromeric transfected *KCNQ2* WT + variants were compared with the currents in *KCNQ2* WT respectively.

[$] The currents in the heteromeric *KCNQ2* WT + *KCNQ3* WT + variants were compared with the currents in *KCNQ2* WT + *KCNQ3* WT respectively.
